# Acetylation in Ionic Liquids Dramatically Increases Yield in the Glycosyl Composition and Linkage Analysis of Insoluble and Acidic Polysaccharides

**DOI:** 10.1021/acs.analchem.3c02056

**Published:** 2023-08-18

**Authors:** Ian M. Black, Ikenna E. Ndukwe, Jiri Vlach, Jason Backe, Breeanna R. Urbanowicz, Christian Heiss, Parastoo Azadi

**Affiliations:** Complex Carbohydrate Research Center, University of Georgia, 315 Riverbend Road, Athens, Georgia 30602, United States

## Abstract

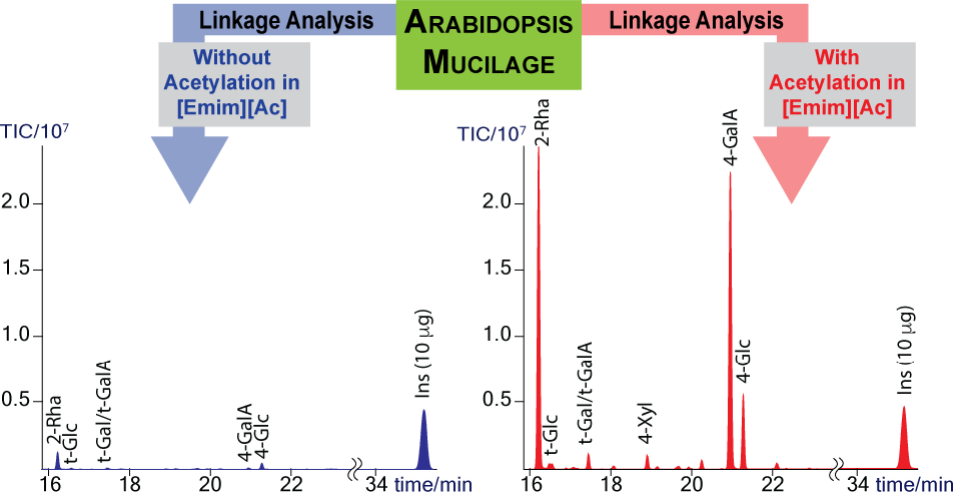

Glycosyl composition and linkage analyses are important first steps toward understanding the structural diversity and biological importance of polysaccharides. Failure to fully solubilize samples prior to analysis results in the generation of incomplete and poor-quality composition and linkage data by gas chromatography–mass spectrometry (GC-MS). Acidic polysaccharides also do not give accurate linkage results, because they are poorly soluble in DMSO and tend to undergo β-elimination during permethylation. Ionic liquids can solubilize polysaccharides, improving their derivatization and extraction for analysis. We show that water-insoluble polysaccharides become much more amenable to chemical analysis by first acetylating them in an ionic liquid. Once acetylated, these polysaccharides, having been deprived of their intermolecular hydrogen bonds, are hydrolyzed more readily for glycosyl composition analysis or methylated more efficiently for glycosyl linkage analysis. Acetylation in an ionic liquid greatly improves composition analysis of insoluble polysaccharides when compared to analysis without acetylation, enabling complete composition determination of normally recalcitrant polysaccharides. We also present a protocol for uronic acid linkage analysis that incorporates this preacetylation step. This protocol produces partially methylated alditol acetate derivatives in high yield with minimal β-elimination and gives sensitive linkage results for acidic polysaccharides that more accurately reflect the structures being analyzed. We use important plant polysaccharides to show that the preacetylation step leads to superior results compared to traditional methodologies.

Polysaccharides are important biomolecules that constitute the primary structural component of plant^1^ and fungal cell walls,^2^ providing mechanical strength and enabling cellular interactions. Polysaccharides also constitute important molecules in bacterial cell walls,^3^ mediating host–cell interactions and maintaining cell shape and structural integrity. In addition to these biological roles, polysaccharides serve important commercial functions and are frequently used as food additives,^4,5^ biofuel feedstocks,^6^ and in biomedical applications^7^ to name a few.

Given the biological importance of polysaccharides in both academic and commercial settings, much attention has been focused on the development of improved analytical techniques to elucidate the structures of these polymers. However, this task is complicated by the tremendous structural diversity present in polysaccharides.^8,9^ First, this complexity is a product of the high number of chiral centers in monosaccharides, which leads to a large number of isomers. In addition, there exist numerous functional groups that can be present through synthetic or biological derivatization, generating even more structural and biological diversity.^10,11^ Finally, their recalcitrant behavior toward both solvolysis and hydrolysis makes the development of improved analytical methods of utmost importance.

Compositional analysis is a basic analytical tool employed to investigate the monosaccharide building blocks of polysaccharides. There are numerous methods utilized for polysaccharide composition analysis, including those that involve colorimetric assays that provide total carbohydrate percentages,^12−14^ methods involving liquid chromatography (LC),^15−17^ and those based on gas chromatography (GC).^18^ In general, the polysaccharide is first depolymerized by acid hydrolysis or methanolysis. The resulting monosaccharides can be directly analyzed by high-pH anion exchange chromatography with pulsed amperometric detection or fluorescently tagged and analyzed by capillary electrophoresis (CE) or LC. Alternatively, the monosaccharides can be derivatized with organic substituents such as trimethylsilyl, methyl, or acetyl functional groups to increase volatility for separation by GC and detection by MS. Acidic hydrolysis of the polysaccharide yields a mixture of monosaccharides that constitute the initial polymer. However, sample insolubility and heterogeneity may result in poor or incomplete hydrolysis, resulting in the generation of nonrepresentative data that underestimate the total carbohydrate content or misrepresent the relative abundance of constituent monosaccharides. Often, harsh hydrolysis conditions are employed to overcome the recalcitrance of insoluble polysaccharides. For example, concentrated sulfuric acid (Saeman hydrolysis) can be employed to dissolve and more thoroughly hydrolyze insoluble polysaccharides, leading to improved monosaccharide quantitation.^19^ While useful to some extent, harsh hydrolysis conditions result in the substantial degradation of the more labile monosaccharides that might be present in a sample. As an alternative, our laboratory developed the methyl alditol (MA) method, in which initial methylation was found to decrease the recalcitrance of insoluble cell wall polysaccharides, allowing complete hydrolysis under much milder conditions than those required for the underivatized sample. This resulted in reduced degradation of labile monosaccharides and further improved yields.^20^

The preparation of partially methylated alditol acetates (PMAAs) for linkage analysis, often referred to as methylation analysis, is another important analytical procedure for polysaccharide analysis. This procedure gives data that can be used to determine the substitution positions of glycosyl residues in an oligosaccharide or polysaccharide. Complete methylation is imperative when performing linkage analysis and interpreting linkage data,^21^ and thus, the methylation step has attracted the most attention in the literature. Methylation requires the generation of alkoxide ions for each free hydroxyl group on the monosaccharides that constitute the polysaccharide, followed by substitution with the methyl group using methyl iodide. Early methods for permethylation of polysaccharides were inefficient and labor-intensive.^22,23^ The difficulty in methylation usually originates in the initial step of alkoxide formation. These earlier methods were eventually replaced by those using methylsulfinyl carbanion (dimsyl anion),^24^ followed by the more modern use of sodium hydroxide, either in the form of ground solid pellets^25^ or in DMSO suspension,^26^ to prepare the polysaccharide alkoxides for subsequent methylation. Traditionally, the use of a dimsyl anion has been recommended when the starting saccharide contains acidic monosaccharides, such as in pectin, to minimize β-elimination reactions.^27,28^ Upon complete methylation, the permethylated sample can be hydrolyzed, the anomeric center reduced with sodium or lithium borodeuteride or lithium aluminum deuteride, and the resulting free hydroxyls, i.e., those that were substituted by another glycosyl residue or involved in formation of the pyranose or furanose ring structure, are acetylated. If the sample does not fully dissolve during the reaction, then the methylation will be incomplete. This will result in poor sample recovery, and the resultant undermethylation will generate artifactual data that could wrongly be interpreted as linkages. In the case of acidic polysaccharides, undermethylation can also result in incomplete carboxyl reduction and the occurrence of β-elimination.

Ionic liquids are salts that exist as a liquid at low temperature (usually defined as <100 °C).^29^ While discovered over 100 years ago,^30^ ionic liquids did not attract significant interest until the last few decades, with numerous papers published on their useful features such as chemically inert, thermally stable, nonflammable, and low-vapor pressure solvents. Ionic liquids have been noted for their utility in diverse applications such as chemical synthesis,^31^ environmental remediation,^32^ and in the industrial sector.^33^ More specifically, research has shown that ionic liquids are capable of dissolving insoluble polysaccharides and lignocellulosic biomass in commercial settings,^34^ with special attention placed on 1,3-dialkylimidazolium acetate-based ionic liquids.^35^ With their ability to dissolve insoluble polysaccharides and thus serve as solvents in derivatization reactions, ionic liquids offer the opportunity to generate polysaccharides with functionalities that render them more amenable to analytical methods that have been traditionally hampered by sample insolubility. Acetylation reactions are of particular interest, as acetylation of polysaccharides results in the addition of a labile functional group that can be easily removed in acidic or basic conditions. Also, acetylated samples become more soluble in organic solvents, such as methanol or dimethyl sulfoxide (DMSO), than their corresponding native counterparts. Protocols for the acetylation of both monosaccharides and polysaccharides using 1-methylimidazole are established.^36,37^ Several reports of acetylation of polysaccharides in ionic liquids have been published.^38−43^ Furthermore, polysaccharides dissolved in 1,3-dialkylimidazolium acetate were shown to be acetylated, even in the absence of a catalyst, as a result of impurities in the solvent.^44^ We chose 1-ethyl-3-methylimidazolium acetate [Emim][Ac] because of its low cost and demonstrated effectiveness in polysaccharide acetylation^35,45,46^

While acetylation in ionic liquids has been applied to obtain solution-state NMR spectra of plant cell walls,^43^ it has never been used to address the challenges arising from the lack of solubility in glycosyl composition analysis. In this paper, we demonstrate for the first time that initial acetylation of polysaccharides in [Emim][Ac] enables the effective and accurate composition analysis of insoluble and soluble polysaccharides simultaneously, an object that has not been achieved satisfactorily with any method to date. Preacetylation in ionic liquids is also shown to drastically improve the linkage analysis of uronic acid-rich polysaccharides, featuring increased yields, more accurate results, and low extent of β-elimination, allowing for the first time reliable determination of linkages of acidic polysaccharides.

## Experimental Section

### Saccharide Materials

Nonadherent and adherent mucilage samples from the seed coats of *Arabidopsis thaliana* were prepared in water by gentle shaking or by ultrasonic treatment, respectively, according to previously published protocols.^47^ Fractions were separately pooled, dialyzed (3500 Da MWCO) against deionized water, and freeze-dried. Mucilage extraction was monitored by microscopic observation after staining with 0.01% (w/v) ruthenium red (Sigma-Aldrich, USA). Celery rhamnogalacturonan-I (RGI) was a gift from Malcolm O’Neill, and BESC standard poplar was obtained from the laboratory of Dr. Debra Mohnen at the University of Georgia.^48^ Potato rhamnogalacturonan I (RGI) was purchased commercially (Megazyme, Ireland). Avicel cellulose (Dupont, USA) and chitin (Sigma, USA isolated from shrimp shells) were obtained commercially.

### Acetylation of Polysaccharides

For the analysis, the sample material (200–400 μg) was dissolved overnight at ambient temperature in 400 μL of [Emim][Ac] with magnetic stirring and treated with 300 μL of acetic anhydride and 50 μL of 1-methylimidazole. After 10 min of stirring, 2 mL of water was added to quench the reaction.

### Isolation of Acetylated Polysaccharides

Acetylated neutral polysaccharides were extracted with 2 mL of dichloromethane (DCM), and the DCM extract was washed with water five times, followed by evaporation of the DCM. Acidic acetylated polysaccharides were dialyzed (1000 Da MWCO) against three changes of deionized water. See the Supporting Information for a detailed procedure.

### TMS Composition Analysis

For the analysis of both the native and acetylated samples, the samples were methanolyzed in 300 μL of 1 M methanolic HCl for 18 h at 80 °C. Amino sugars were re-N-acetylated in 400 μL of 2:1:1 methanol-pyridine-acetic anhydride (15 min at ambient temperature). Hydroxyls were then per-O-trimethylsilyl (TMS) derivatized as previously described.^49^ See Supporting Information for a detailed procedure.

### Preparation of PMAAs from Acidic Polysaccharides

The acetylated samples were dissolved in 300 μL of dimethyl sulfoxide (DMSO), 300 μL dimsyl base^50^ was added, and the mixture was cooled in ice before dropwise addition of 400 μL ice-cold iodomethane. The mixture was removed from the ice bath and stirred for 2 h, after which time 2 mL of water followed by 2 mL of DCM was added. The organic layer was separated and washed with water five times, and the DCM was evaporated. The carboxylic esters were reduced with 5 mg/mL lithium aluminum deuteride in 300 μL of THF at 80 °C for 4–6 h. The mixture was acidified by the addition of 100 μL of 9:1 ethanol/acetic acid and evaporated. The sample was dissolved in 500 μL of water and dialyzed (1000 Da MWCO). The dialyzed sample was remethylated with NaOH suspension as previously described,^26^ extracted with DCM, hydrolyzed with 300 μL of 2 M trifluoroacetic acid (TFA), reduced with 10 mg/mL sodium borodeuteride and, after removal of borate by drying with 9:1 ethanol/acetic acid, acetylated with 250 μL of acetic anhydride and 230 μL of concentrated TFA. See the Supporting Information for a detailed procedure.

### GC-MS Analysis

GC-MS analysis of the TMS methyl glycosides and partially methylated alditol acetates (PMAAs) was performed using an Agilent 7890A GC interfaced to a 5975C MSD. The composition analysis employed a Supelco Equity-1 fused silica capillary column (30 m × 0.25 mm ID), while the linkage analysis used a Supelco SP-2330 fused silica capillary column (30 m × 0.25 mm ID). More details are found in the Supporting Information.

### NMR Spectroscopy

One- and two-dimensional NMR spectra (^1^H–^1^H COSY, TOCSY, ROESY, and ^1^H–^13^C HSQC and HMBC) were acquired on a 2 mg sample in CDCl_3_ at 25 °C on a Varian VNMRS spectrometer (^1^H, 599.66 MHz) equipped with an inverse triple-resonance HCN cold probe. ^1^H and ^13^C chemical shifts were referenced to the respective signals of residual CHCl_3_ and bulk CDCl_3_ at 7.26 and 77.2 ppm. NMR data were processed and analyzed in MestreNova (version 14.2.3). More details are found in the Supporting Information.

## Results and Discussion

### Acetylation of Polysaccharides in [Emim][Ac]

Acetylation of monosaccharides and partially methylated polysaccharides had previously been accomplished using acetic anhydride and 1-methylimidazole.^37,51^ Acetylation has also been accomplished in ionic liquids.^38−43^ We found [Emim][Ac] to be a good solvent for the acetylation of polysaccharides. For this purpose, the samples were first dissolved completely in [Emim][Ac] by stirring overnight at room temperature. The subsequent acetylation took place at room temperature and was fast, with reaction times of only 10 min. We found that it was necessary to adapt the cleanup procedures depending on whether the sample contained neutral or acidic sugars because the recovery using a simple DCM extraction was ineffective for acidic polysaccharides. Neutral polysaccharides could be extracted by using DCM, but acidic polysaccharides required dialysis. It is important to limit the reaction time for the acetylation to 10 min or less to avoid a deep red color formation and concomitant appearance of large, noncarbohydrate peaks in the GC chromatogram during the composition analysis.

### Comparison of Composition Analysis of Insoluble Polysaccharide Standards

**Figure 1:**
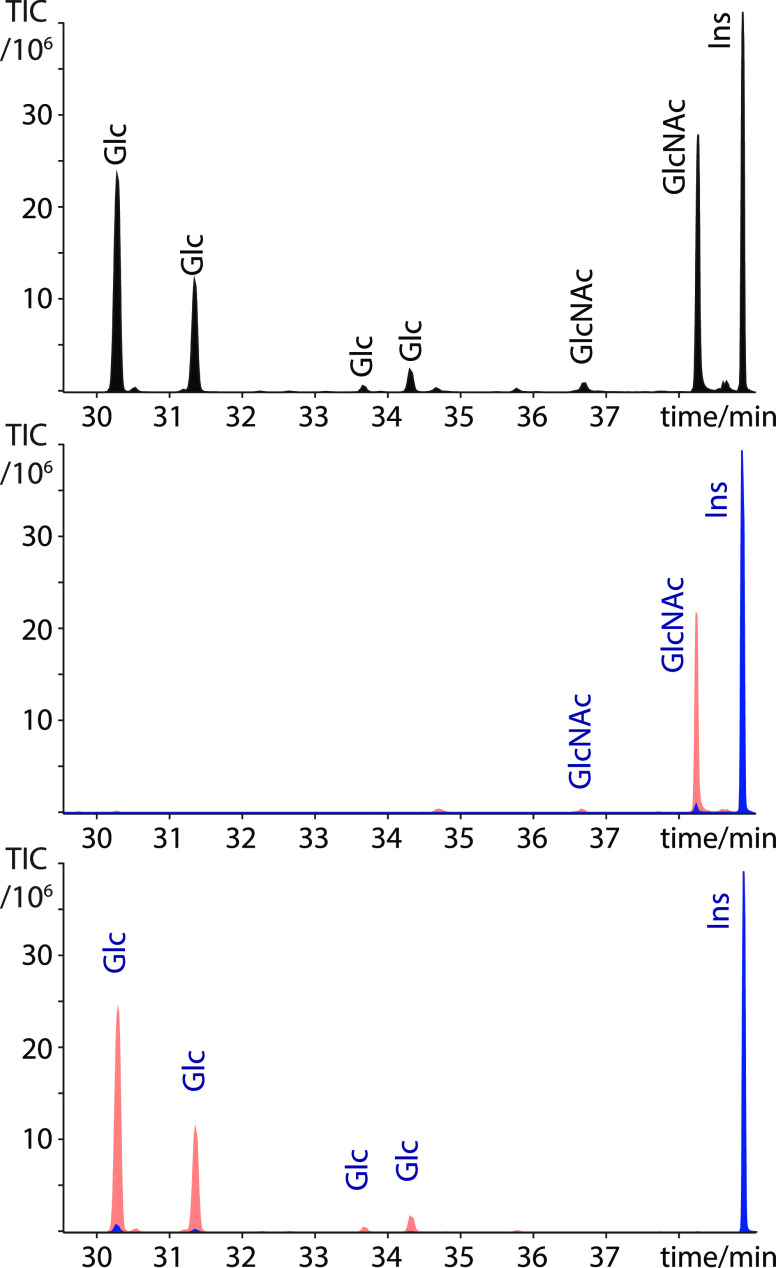
Gas chromatograms of the TMS methyl glycosides obtained from 50 μg of glucose and 50 μg of GlcNAc (top) and composition analysis results of 50 μg of chitin (middle) and 50 μg of cellulose (bottom), with (red trace) and without (blue trace) preacetylation. All samples contained 20 μg of *myo*-inositol as an internal standard. Quantitation of the monosaccharides is given in Table 1.

The utility of preacetylation was tested for composition analysis of insoluble polysaccharides using cellulose and chitin as examples. Both polysaccharides represent key structural components of plant and fungal cell walls, respectively. To evaluate the effectiveness of acetylation, we compared the glycosyl composition analysis results of these polysaccharides without and with preacetylation. The glycosyl composition analysis was performed by GC-MS of trimethylsilyl (TMS) methyl glycosides, prepared from the polysaccharides according to the well-established protocol.^18^ The results of this analysis, along with the monosaccharide standards used for quantitation, are shown in Figure 1 and Table 1. The middle and bottom panels of Figure 1 show the overlaid chromatograms for the chitin and cellulose samples, respectively. Both sets of overlaid samples are normalized to *myo*-inositol; the same amount of *myo*-inositol was added to all samples. In both samples, those without preacetylation predictably gave low-intensity peaks (glucose for cellulose, N-acetylglucosamine for chitin), indicating that only a small portion of the samples was methanolyzed during the analysis (blue traces). In both cases, the preacetylated samples (red traces) showed much larger peaks, demonstrating that preacetylation resulted in a much better recovery of glucose (Glc) from cellulose and N-acetylglucosamine (GlcNAc) from chitin. Table 1 shows recoveries of only 1 or 2% for the samples without preacetylation, while recovery for the preacetylated samples was much better (70% for chitin and 90% for cellulose).

**Table 1:** Determined Mass and Recovery (in % w/w) of the Monosaccharides Obtained from Cellulose and Chitin with and without Pre-acetylation in the Ionic Liquid in Figure 1

polymer	product	mass (recovery)	
		native	preacetylated
50 μg of cellulose	glucose	1.4 μg (1.3%)	50.0 μg (90%)
50 μg of chitin	GlcNAc	1.4 μg (2.6%)	38.1 μg (70%)

### Comparison of Composition Analysis of Poplar Wood with and without Acetylation

**Figure 2:**
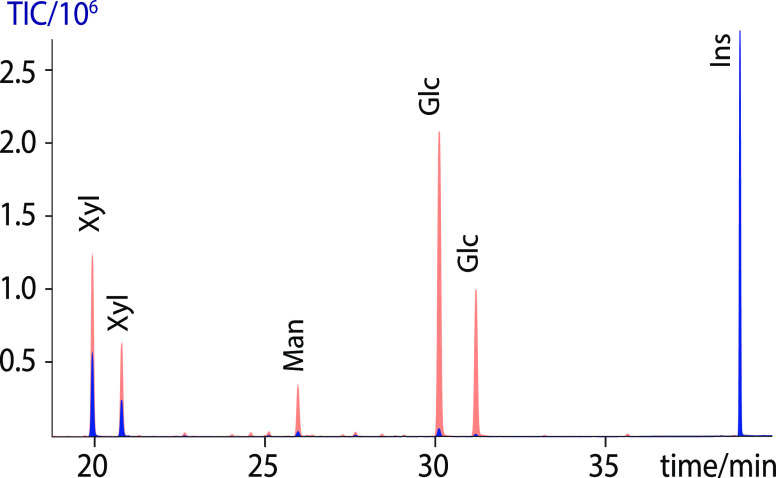
Comparison of the TMS methyl glycoside composition analysis of equal amounts of BESC poplar wood without (blue) and with acetylation (red trace). Quantitation of the monosaccharides is shown in Table 2. The chromatograms are normalized to inositol.

We next sought to compare the acetylation of a more complex, commercially relevant sample containing a mixture of soluble and insoluble polysaccharides. We chose Bioenergy Science Center (BESC) standard poplar wood,^48^ as poplar is considered an important biofuel feedstock and known to contain significant amounts of insoluble cellulose. We performed composition analysis on the woody cell wall material with and without the preacetylation. The results of this analysis indicated a composition of the native poplar sample (Figure 2, blue trace) consisting mainly of xylose residues, with only a small amount of mannose and glucose present (Figure 2 and Table 2), suggesting that the soluble hemicellulose xylan is over-represented using this technique. When the composition analysis was performed after preacetylation (Figure 2, red trace), the sample was found to contain mainly glucose (from cellulose) along with xylose (xylan) and mannose (mannan).

**Table 2:** Determined Carbohydrate Amount and Monosaccharide Composition (in mol %) of the Poplar Samples (∼280 μg Each) Shown in Figure 2

glycosyl residue	mass (mol %)	
	native	preacetylated
Xylose (Xyl)	23.2 μg (89.8%)	54.5 μg (32.9%)
Mannose (Man)	0.7 μg (2.2%)	6.8 μg (3.4%)
Glucose (Glc)	2.5 μg (8.0%)	126.8 μg (63.7%)
Total	26.4 μg	188.2 μg

The estimation of each monosaccharide (Table 2) shows that the preacetylation reaction has resulted in significantly higher recovery of all three of the monosaccharides present in the sample and increased the overall estimation of carbohydrate in the samples from 9 to 70%. These data, along with the previous results, show that dissolving insoluble polysaccharide samples in ionic liquids and then preacetylating them prior to composition analysis give results that are consistent with significantly increased yields of the insoluble polysaccharide components.

### Comparison of Composition Analysis of Soluble RGI

**Figure 3:**
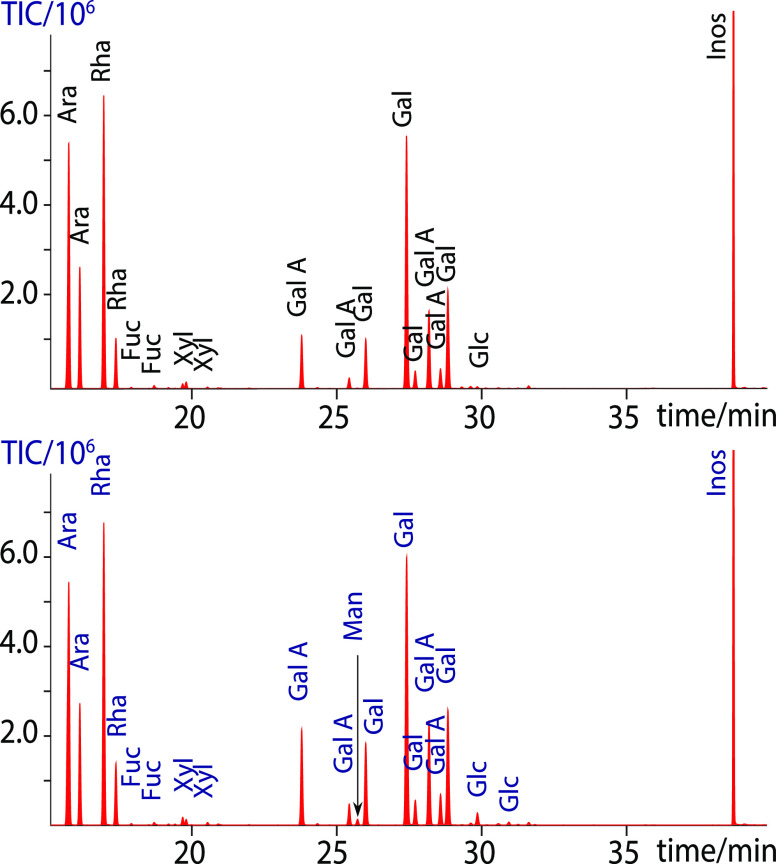
Comparison of the TMS methyl glycoside composition analysis of celery RGI with (bottom) and without preacetylation (top).

**Figure 4:**
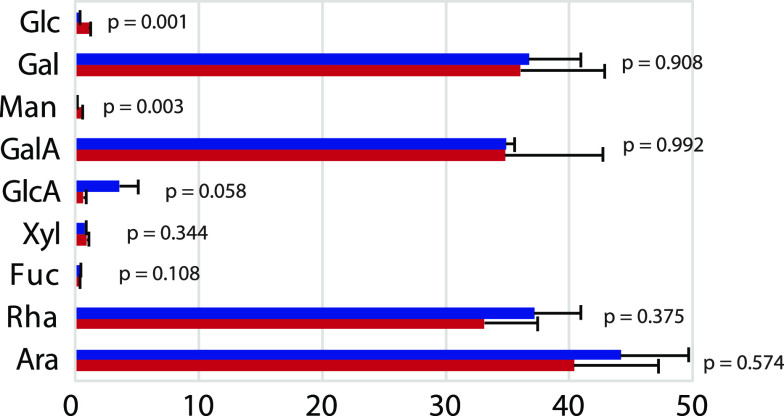
Comparison of the estimated celery RGI composition results with (red) and without (blue) preacetylation as seen in Figure 3. Data represent averages of the triplicate analysis. With the exception of the minor residues glucose and mannose, the differences between the analyses are not significant (*p* > 0.05).

We compared the composition results of RGI, an anionic polysaccharide with a backbone comprising a disaccharide repeating unit of galacturonic acid and rhamnose in a 1:1 ratio^52^ and side chains mostly composed of arabinose and galactose. We performed composition analysis with and without preacetylation to determine whether this procedure works also for soluble polysaccharides. We carried out the analysis in triplicate of 200 μg of RGI isolated from celery. Representative chromatograms of these analyses are shown in Figure 3. The averaged mass recoveries were not significantly different between preacetylated and nonacetylated samples (Figure 4), demonstrating that the composition analysis of soluble polysaccharides is not affected by the preacetylation procedure and confirming that the preacetylation protocol can be used on complex samples containing both soluble and insoluble polysaccharides.

When we performed the linkage analysis with and without preacetylation on potato RGI, we found that the preacetylation uncovered the presence of a small amount of hemicellulose contamination, as evidenced by nonpectic monosaccharides being present in the composition analysis of this sample (Figure S1). This hemicellulose contamination likely accounts for the incomplete solubility of potato RGI in aqueous solution

### Preacetylation Improves Recovery and Linkage Results of Mucilage

**Figure 5:**
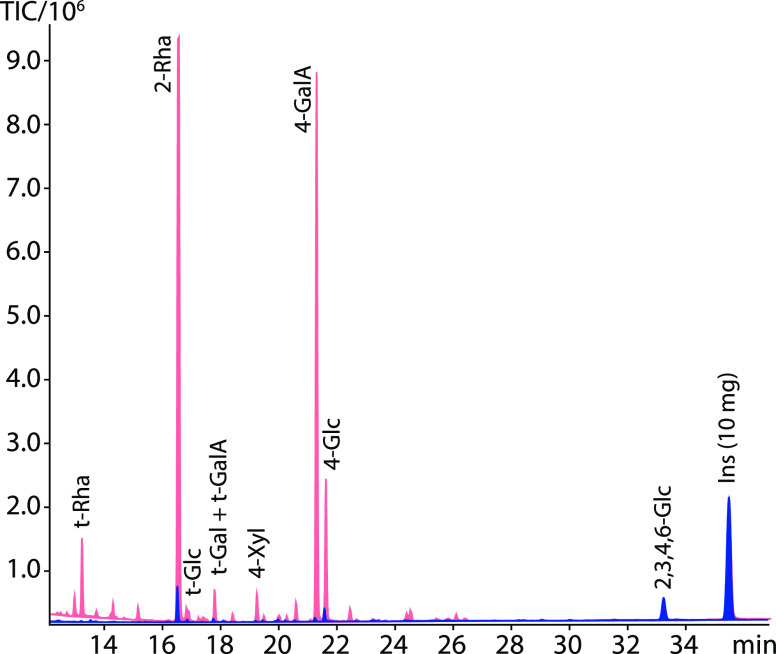
Linkage analysis results generated from the PMAAs of the nonadherent *Arabidopsis* seed mucilage without (blue trace) and with (red trace) preacetylation, showing that preacetylation leads to 10-fold increase in 2-Rha and 100-fold increase in 4-GlcA recovery, as well as ∼1:1 ratio between these residues, in agreement with the published structure.^52^ The 2,3,4,6-Glc residue is a result of undermethylation, which was only observed in the nonacetylated analysis.

**Figure 6:**
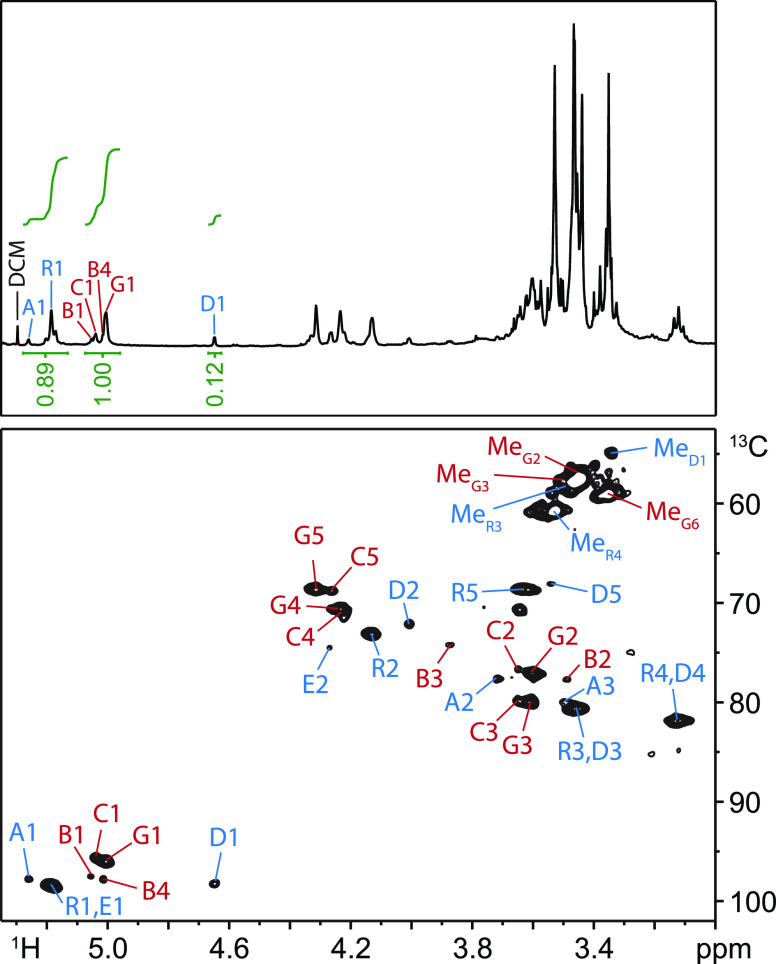
Partial one-dimensional ^1^H NMR spectrum (top) and partial two-dimensional ^1^H–^13^C-HSQC NMR spectrum (bottom) of methylated and carboxyl-reduced nonadherent *Arabidopsis* seed mucilage. Anomeric carbons derived from rhamnose (A1, R1, E1, D1) and galacturonic acid (B1, C1, G1) are equal in abundance, as evidenced by a 1:1 ratio of integrals (full peak assignment in Table S1). Residue B, resulting from β-elimination amounts to <8% of GalA residues.

Nonadherent seed mucilage from *Arabidopsis thaliana* is composed primarily of RGI backbone with equal abundance of rhamnose and galacturonic acid.^52^ However, conventional linkage of this material consistently underestimates the ratio of 4-linked galacturonic acid to 2-linked rhamnose and is characterized by an overall low recovery (Figure 5, blue trace). We suspected that this was the result of the poor solubility of the highly anionic polysaccharides in DMSO, as well as β-elimination of galacturonic acid during the initial methylation step. We reasoned that preacetylation of the sample might increase solubility and uronic acid recovery. To test this hypothesis, the mucilage sample was preacetylated, then permethylated, carboxyl reduced, and remethylated. One-dimensional ^1^H and two-dimensional ^1^H–^13^C-heteronuclear single quantum coherence (HSQC) NMR showed that the methylation was successful, with only 8% of β-elimination products (Figure 6, Table S1) and a 1:1 molar ratio of galacturonic acid and rhamnose anomeric protons, in accordance with the published glycosyl composition of *Arabidopsis* mucilage.^52^ We then prepared the partially methylated alditol acetate (PMAA) derivatives of the methylated mucilage sample by hydrolysis, reduction, and acetylation of the partially methylated alditols. Analysis of the PMAAs confirmed that the galacturonic acid and rhamnose linkages were present in a 1:0.9 ratio (Figure 5, Table 3), similar to that observed by NMR spectroscopy, the slight reduction of rhamnose likely being due to higher volatility of its PMAA. Overall, the recovery of 2-Rha was 10 times higher, and that of 4-GalA 100 times higher than in the traditional linkage analysis. Close inspection of the gas chromatograms also gave information on the other polysaccharides present in the mucilage in minor amounts. These results strongly indicated that the preacetylation greatly benefits the linkage analysis of uronic acid-containing polysaccharides.

**Table 3:** Percentage of Each Linkage Residue Relative to the Sum of Linkage Residues Detected in the Nonadherent Mucilage after O-Acetylation (cf. Figure 5)

residue	% area
terminal rhamnopyranosyl (t-Rha)	2.2
terminal arabinofuranosyl (t-Araf)	0.3
terminal xylopyranosyl (t-Xyl)	0.5
2-linked rhamnopyranosyl (2-Rha)	35.8
terminal glucopyranosyl (t-Glc)	0.5
terminal galactopyranosyl (t-Gal)	1.0
terminal galactopyranosyl Uronic Acid (t-GalA)	0.7
5-linked arabinofuranosyl (5-Araf)	0.6
4-linked xylopyranosyl (4-Xyl)	1.6
2,3-linked rhamnopyranosyl (2,3-Rha)	0.4
2,4-linked rhamnopyranosyl (2,4-Rha)	0.6
4-linked rannopyranosyl (4-Man)	1.3
4-linked galactopyranosyl uronic acid (4-GalA)	41.0
4-linked glucopyranosyl (4-Glc)	11.1
2,4-linked xylopyranosyl (2,4-Xyl)	0.6
3,4-linked galactopyranosyl uronic acid (3,4-GalA)	0.5
2,4-linked galactopyranosyl uronic acid (2,4-GalA)	0.5
4,6-linked mannopyranosyl (4,6-Man)	0.8

### Comparison of Linkage Protocols Demonstrates the Utility of the Improved Methylation and Linkage Analysis Results

**Figure 7:**
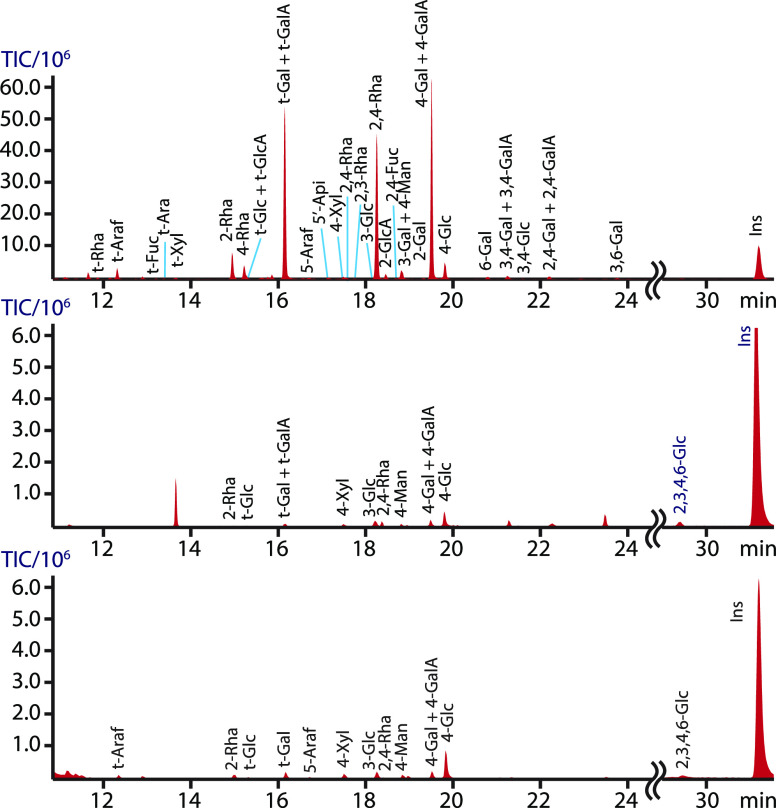
Total ion chromatogram of the PMAAs generated from potato RGI using different methylation protocols. Equivalent amounts of potato RGI were either acetylated and methylated with dimsyl (top) or not acetylated and methylated with dimsyl (middle) or sodium hydroxide (bottom). The comparison shows drastic improvement in the intensity of carbohydrate peaks and the number of linkages present due to preacetylation. In addition, the 2,3,4,6-Glc artifact arising from incomplete methylation in the samples without acetylation was not detected in the acetylated sample. The bottom and middle chromatograms are expanded 10× to show the minor peaks.

To further test the effects of acetylation on the derivatization of more complex, branched polysaccharides, we performed methylation analysis of potato RGI with and without preacetylation. The preacetylated sample was methylated using dimsyl base, while samples without preacetylation were methylated using either dimsyl base or sodium hydroxide. To gauge the effectiveness of these different methylation protocols, the sample (with and without preacetylation) was permethylated (with either NaOH or dimsyl), carboxyl reduced, remethylated (with NaOH), and then converted to PMAA derivatives for analysis. Inositol (10 μg) was added to each sample as an internal standard after the second methylation step. The internal standard enables comparison of the peak intensities of the various glycosyl residues to determine if one method results in significantly better yields than the others. These results are listed in Figure 7 and Table 4. As with the mucilage results above, we found that linkage analysis of this more complex, branched RGI also benefited from preacetylating the sample prior to methylation. The data for the potato RGI showed significant improvement when preacetylated prior to permethylation (Figure 7, top), compared to either the dimsyl (middle) or sodium hydroxide (bottom) treated samples without preacetylation. The preacetylated sample contained significantly more peaks, and the peak intensity was greatly increased, as evidenced by the comparison of the linkage residues to inositol. Also, the amount of 4-linked galacturonic acid was notably increased (Table 4), suggesting that the uronic acid recovery was enhanced by preacetylation. Furthermore, the absence of the 2,3,4,6-Glc residue in the acetylated sample indicated complete permethylation, in contrast to the nonacetylated samples. Thus, highly substituted potato RGI showed substantially better recovery and derivatization efficiency when the sample was acetylated prior to methylation (Figure 7). This included not only total recovery but also the ratio of sugars recovered.

**Table 4:** Percentage of Each Linkage Residue Relative to the Sum of Linkage Residues Detected in the Potato RGI Samples after Different Methylation Protocols

residue	pre-acetylated	native	
	dimsyl	NaOH	dimsyl
t-Rha	0.3		
t-Ara*f*	1.9		4.8
t-Fuc	0.1		
t-Ara	0.1		
t-Xyl	0.4		
2-Rha	4.8	1.6	5.0
4-Rha	2.5		
t-Glc	0.3	3.3	2.3
t-GlcA	0.1		
t-Gal	23.1	10.2	10.0
t-GalA	2.0	0.7	
5-Ara*f*	0.4		2.7
5′-Api*f*	0.4		
4-Xyl	0.5	7.0	8.1
3,4-Rha	0.2		
2,3-Rha	0.1		
3-Glc	-	7.7	4.8
2,4-Rha	23.3	9.3	6.2
2-GlcA	0.9		
2,4-Fuc	0.2		
3-Gal	1.1		
4-Man	1.0	7.4	6.1
2-Gal	0.2		
4-Gal	7.7	4.0	2.6
4-GalA	22.0	9.8	7.5
4-Glc	3.2	27.1	40.1
6-Gal	0.8		
3,4-Gal	0.4		
3,4-GalA	0.6		
3,4-Glc	0.1		
2,4-Gal	0.4		
2,4-GalA	0.5		
3,6-Gal	0.4		
2,3,4,6-Glc		11.8	5.2

## Conclusions

Our work has shown that acetylation in ionic liquids provides a new and effective way to achieve the accurate and comprehensive glycosyl composition analysis of recalcitrant biomass through high-yielding chemical derivatization. The protocol is a relatively short and simple method for polysaccharide acetylation and demonstrates that the preacetylation yields polysaccharides more amenable to analysis. Preacetylation leads to a more complete hydrolysis of insoluble polysaccharides such as cellulose and chitin, without degrading or otherwise negatively affecting composition results. We demonstrate this by comparing composition analysis of highly insoluble poplar wood with and without preacetylation. We further show that preacetylation benefits linkage analysis of highly acidic polysaccharides, resulting in better recovery and generating results more in agreement with expected structures. We believe that this method will be especially valuable for complex mixtures containing both soluble and insoluble polysaccharides, such as plant cell walls comprising cellulose and hemicellulose. It will also aid in the analysis of polysaccharides containing less stable monosaccharide residues, which will be better preserved due to the mild hydrolysis conditions made possible by acetylating the samples.

## Supporting Information Available

Detailed protocols for the composition analysis of insoluble polysaccharides and linkage analysis of acidic polysaccharides. Experimental details for the NMR analysis of permethylated *Arabidopsis* seed mucilage and NMR assignments. (PDF)
