# Supplementary material for: Acetylation in Ionic Liquids Dramatically Increases Yield in the Glycosyl Composition and Linkage Analysis of Insoluble and Acidic Polysaccharides
Source: Anal Chem. 2023 Aug 18;95(34):12851–8. doi: 10.1021/acs.analchem.3c02056 (PMC10469378; doi:10.1021/acs.analchem.3c02056)
Supplement: Supplementary file 1 — ac3c02056_si_001.pdf [file ac3c02056_si_001.pdf]

Supporting Information

Acetylation in ionic liquid dramatically increases yield in glycosyl composition and linkage analysis of insoluble and acidic polysaccharides

Ian M. Black,[1] Ikenna E. Ndukwe,[1,2] Jiri Vlach,[1] Jason Backe,[1] Breeanna R. Urbanowicz,[1] Christian Heiss,[1,*] Parastoo Azadi.[1]

[1]Complex Carbohydrate Research Center, University of Georgia, 315 Riverbend Road, Athens Ga, 30602, USA

[2]Current address: Pivotal Attribute Sciences, Amgen Inc., One Amgen Center Drive, Thousand Oaks, CA 91320

Table of Contents

Protocol for the Composition Analysis of Insoluble Polysaccharides S1

Protocol for the Generation of Partially Methylated Alditol Acetates (PMAAs) of Uronic Acid-Containing Samples S3

GC-MS Analysis S6

Notes to Protocols S7

NMR Spectroscopy Experimental Details S8

Figure S1 S8

NMR Assignments S9

Notes to NMR Table S9

References S10

Protocol for the Composition Analysis of Insoluble Polysaccharides

Acetylation of Sample

1 – Weigh out 200 – 400 μg of sample in a clean test tube fitted with a Teflon lined screw cap.

2 – Add 400 μl dry 1-ethyl-3-methylimidazolium acetate ([Emim][Ac]) to the sample along with a stir bar (Note 1).

3 – Stir sample overnight at room temperature to fully dissolve.

4 – Add 300 μl acetic anhydride and 50 μl N-methylimidazole to the sample.

5 – Stir for 10 minutes at room temperature. Add 2 mL water to quench reaction (Note 2).

Extraction For sample Containing Significant Amounts of Uronic Acids (Note 3)

6a – Transfer sample to 1000 Da MWCO dialysis tubing (regenerated cellulose) and dialyze against DI water for 2 days, replacing the water a minimum of three times to remove the ionic liquid.

7a – Transfer the dialyzed sample back into a test tube, taking care to recover as much of the sample from the dialysis tube as possible.

8a – Add 20 µg of inositol to the sample and lyophilize.

Extraction For Samples Containing Neutral Sugars

6b – Add 2 mL dichloromethane (DCM) to the sample.

7b – Vortex for 5 seconds, then separate the layers via low-speed centrifugation for 5 seconds.

8b – Decant the top aqueous layer and replace with 2 mL fresh DI water.

9b – Repeat vortexing and centrifugation steps for a total of 5 sample washes.

10b – On the last wash step, decant the aqueous layer and evaporate DCM.

11b – Add 20 µg inositol to the sample and lyophilize to remove any remaining water.

TMS Composition Analysis of Acetylated Samples

1 – To the lyophilized sample, add 300 μl 1M methanolic HCl.

2 – Heat 16–18 hours at 80 ºC.

3 – Dry the sample completely by a stream of nitrogen or dry air, add 200 μl methanol (MeOH)and dry again. Repeat the MeOH addition and drying for a total of 3 times.

4 – Samples containing amino sugars require a re-N-acetylation step (Note 4). Add 200 µl MeOH, 100 µl pyridine and 100 µl acetic anhydride to the dry sample. Let the sample react for 15 minutes at room temperature and dry the reaction mixture completely.

5 – Add 300 µl TMS reagent (Tri-Sil HTP reagent, Thermo) to the dry sample and heat to 80 ^{o}C for 20 minutes.

6 – Gently dry down TMS reagent until approximately 50 µl are left.

7 – Add ~250 µl hexane to the sample and centrifuge to pellet the insoluble TMS reaction byproducts.

8 – Transfer the supernatant to a new test tube and dry nearly completely (Note 5).

9 – Add ~200 µl hexane and transfer to a GC vial insert.

Protocol for the Generation of Partially Methylated Alditol Acetates (PMAAs) of Uronic Acid-Containing Samples

Acetylation

1 – Weigh out 500 – 1000 µg of sample in a clean test tube fitted with a Teflon-lined screw cap.

2 – Add ~300 µl dry [Emim][Ac] to the sample along with a stir bar (Note 1).

3 – Stir sample overnight at room temperature to fully dissolve.

4 – Add 300 µl acetic anhydride and 50 µl N-methylimidazole to the sample.

5 – Stir for 10 minutes at room temperature. Add 1 mL water to quench reaction (Note 2).

6 – Transfer sample to dialysis tubing and dialyze against DI water for 2 days, replacing the water a minimum of three times to remove the ionic liquid.

7 – Transfer the dialyzed sample back into the test tube, taking care to recover as much of the sample from the dialysis tube as possible and lyophilize.

First Methylation

1 – Add ~300 µl dimethyl sulfoxide (DMSO) and a stir bar to the sample and stir overnight to dissolve.

2 – Add ~300 µl prepared dimsyl base to the sample and stir for ~ 2 hours (Note 6).

3 – Freeze the sample by submerging it in a beaker of ice water.

4 – Add ~400 µl ice cold iodomethane dropwise to the sample (Note 7).

5 – Allow the sample to stir for ~2 hours.

6 – Add 2 mL of water to the sample, followed by 2 mL of DCM.

7 – Vortex the sample for ~5 seconds, then separate the layers via low-speed centrifugation for ~5 seconds.

8 – Remove the top aqueous layer and discard. Replace with 2 mL of fresh water (Note 8).

9 – Repeat the water wash step for a total of 5 times.

10 – Remove the aqueous layer one final time and evaporate the sample to dryness. Lyophilize to ensure the sample is completely dry.

Reduction of the Uronic Acid Methyl Esters

1 – Add ~300 μl of a 5 mg/mL solution of lithium aluminum deuteride ($LiAlD_4$) in dry tetrahydrofuran (THF) to the sample (Note 9).

2 – Heat at 80 °C for 4–6 hours.

3 – Add 100 μl 9:1 MeOH:acetic acid to acidify the sample (Note 10).

4 – Dry the solvents. Add another 100 μl of 9:1 MeOH:acetic acid and dry again.

5 – Add 500 μl of water to dissolve the sample and salts and transfer to a 1000 Da MWCO dialysis bag. Vortex the sample tube twice more with water and combine all in the dialysis bag.

6 – Dialyze the sample against DI water for 2 days, replacing the water a minimum of three times to remove the salts generated by the neutralization of $LiAlD_4$ with acetic acid.

7 – Transfer the dialyzed sample back into the test tube, taking care to recover as much of the sample from the dialysis tube as possible.

Second Methylation

1 – Add ~300 μl DMSO and a stir bar to the sample and stir overnight to dissolve.

2 – Add ~300 μl of sodium hydroxide suspension in DMSO to the sample and stir at room temperature for 15 minutes (Note 11).

3 – Add 100 μl iodomethane to the sample and stir approximately 25 minutes.

4 – Repeat steps 2 and 3 once more.

5 – Add 2 mL of water to the sample, followed by 2 mL of DCM.

6 – Vortex the sample for ~5 seconds, then separate the layers via low-speed centrifugation for ~5 seconds.

7 – Remove the top aqueous layer and discard. Replace with 2 mL of fresh water (Note 12).

8 – Repeat the water wash step for a total of 5 times.

9 – Remove the aqueous layer one final time and dry the sample, lyophilize to ensure the sample is completely dry. Alternatively, the bottom organic layer can be transferred to a new tube and dried.

10 – At this point, 10 ug of inositol can be added to the sample.

Hydrolysis

1 – Add ~300 µl 2 M trifluoroacetic acid (TFA) to the sample and heat in a sealed tube at 120 ^{o}C for 2 hours.

2 – Dry the sample thoroughly to remove the acid. Add 100 µl isopropy lalcohol (IPA) and dry again.

3 – Repeat step 2 twice, for a total of 3 times.

Anomeric Group Reduction

1 – Add 300 µl of a 10 mg/ml solution sodium borodeuteride prepared in 100 mM ammonium hydroxide to the sample.

2 – Allow to react at room temperature for a minimum of 3 hours, preferably overnight.

3 – Add 100 µl 9:1 MeOH:acetic acid to acidify the sample. Dry the sample.

4 – Add another round of 9:1 MeOH:acetic acid and dry again.

5 – Repeat step 4 once more for a total of three times (Note 13).

Acetylation

1 – Add 250 µl acetic anhydride and 230 µl concentrated TFA to the sample.

2 – Heat at 35 ^{o}C for 20 minutes.

3 – Dry down sample until ~100 µl remains.

4 – Add 2 mL of water to the sample, followed by 2 mL of DCM.

5 – Vortex the sample for ~5 seconds, then separate the layers via low-speed centrifugation for ~5 seconds.

6 – Remove the top aqueous layer and discard. Replace with 2 mL of fresh water.

7 – Repeat the water wash step for a total of 5 times.

8 – On the last wash, remove the bottom DCM layer and transfer to a new tube (Note 14).

9 – Dry the DCM layer. Dissolve the dried sample in ~200 µl DCM and transfer to a GC vial insert.

GC-MS Analysis

GC-MS analysis of the TMS methyl glycosides and partially methylated alditol acetates (PMAAs) was performed using an Agilent 7890A GC interfaced to a 5975C MSD. The composition analysis employed a Supelco Equity-1 fused silica capillary column (30 m X 0.25 mm ID), while the linkage analysis used a Supelco SP-2330 fused silica capillary column (30 m X 0.25 mm ID).

For the composition analysis, the GC ramp conditions were as follows: the initial starting temperature was 80 °C, the oven was ramped at a rate of 20 °C per minute until the temperature reached 140 °C and was held for 2 min. After 2 min, the GC was ramped at a rate of 2 °C per minute until it reached 200 °C. Finally, the GC was ramped at a rate of 30 °C per minute until the GC reached a final temperature of 250 °C and held for 5 minutes.

For the linkage analysis, the initial starting temperature was 60 °C, and the oven was ramped at a rate of 27.5 °C per minute until the temperature reached 170 °C. The ramp was then slowed to 4 °C per minute until it reached 235 °C and held at this temperature for 2 minutes. Finally, the GC was ramped at a rate of 3 °C per minute until the GC reached a final temperature of 240 °C and held for 7 minutes.

Quantitation of the sugars was performed by addition of an internal standard (20 µg inositol) to the samples and to a standard set of neutral and acidic sugars (50 µg each) run alongside the samples. From the standards, response factors could be obtained for each individual sugar in the standard. These response factors allowed us to calculate the amounts and subsequent molar percentage of each carbohydrate detected in the samples using their combined peak areas.

Notes to Protocols

1 - [Emim][Ac] is hygroscopic and as such, care should be taken to store the ionic liquid under dry conditions.

2 - The reaction mixture will become increasingly darker the longer the reaction is allowed to go, and the byproducts that are generated will be visible in the GC chromatogram. So, it is important that the reaction is not performed for longer than 10 minutes.

3 - Dialysis is required for samples containing uronic acids, but it can also be employed for neutral sugar analysis. In fact, the dialysis leads to smaller contaminating side products (mentioned in note 2) peaks in the GC chromatogram. But care must be taken to fully recover the insoluble material from the dialysis membrane.

4 – If sample does not contain aminosaccharides, the re-N-acetylation step (Step 4 under the TMS Composition Analysis of Acetylated Samples section) can be skipped.

5 - At this point, the TMS derivatized monosaccharides are volatile, and so care must be taken to not over-dry the sample.

6 – Dimsyl potassium base (potassium methylsulfinylmethylide) preparation is detailed by Harris et al.[1]

7 – The iodomethane reaction with dimsyl base is exothermic. The sample is frozen first to minimize the temperature rise. As iodomethane is added, the sample will heat and melt. The addition should be done while stirring on a stir plate. Often, but not always, the dimsyl is completely quenched by the iodomethane, resulting in a color change of the sample.

8 – Typically there is not much of an interphase layer between the DCM and water layers. The interphase layer indicates insufficient methylation/acetylation of the sample. Interphase material should be retained, and care taken to not remove it with the water layer.

9 – $LiAlD_4$ reacts with residual water, so the THF should be dry. It is recommended to store the THF with molecular sieves or under argon in an airtight container.

10 – Addition of acetic acid will decompose the residual $LiAlD_4$, resulting in a noticeable bubbling as deuterium gas is generated by the reaction. If no bubbling is observed, the initial $LiAlD_4$ reagent in THF may have partly decomposed.

11 – Instructions for preparation of NaOH/DMSO base reagent are detailed by Anumula and Taylor.[2]

12 – In the event that the sample still contains significant interphase material, residual salt leftover from the uronic acid reduction step may have interfered with the second round of methylation, resulting in undermethylation. In this case, the interphase material should be retained through the water washes, and the NaOH methylation reaction should be repeated to ensure complete methylation of the polymer.

13 – A noticeable white crust should appear. These are the salt products of the reducing agent and acetic acid. At this point, the sample is done drying.

14 – Here, it is better to leave some of the DCM layer behind so as not to transfer water with the DCM. Any water present at the end would need to be dried before the final reconstitution of the sample in DCM.

NMR Spectroscopy Experimental Details

Approximately 2 mg of the sample of permethylated and reduced unsubstituted rhamnogalacturonan was dissolved in 550 µL $CDCl_3$ (99.9% D, Sigma) and placed into a 5 mm NMR tube. NMR data were acquired at 25 °C on a Varian VNMRS spectrometer (^1H, 599.66 MHz) equipped with an inverse triple-resonance HCN cold probe. Quantitative ^1H data were acquired with spectral width of 9615 Hz, 16384 data points and four transients with total recycle delay of 30 s between each scan. For the 2D experiments, the indirect and direct ^1H spectral widths were set to 6 ppm, centered at 3 ppm, and ^{13}C spectral width was 70 ppm, centered at 85 ppm. The homonuclear 2D data (COSY, TOCSY, ROESY) were acquired with 160 increments and the heteronuclear 2D data (HSQC, HMBC) with 128 increments. The TOCSY and ROESY spectra were both acquired with mixing time of 80 ms. ^1H and ^{13}C chemical shifts were referenced to the respective signals of residual $CHCl_3$ and bulk $CDCl_3$ at 7.26 and 77.2 ppm. NMR data were processed and analyzed in MestreNova (version 14.2.3).

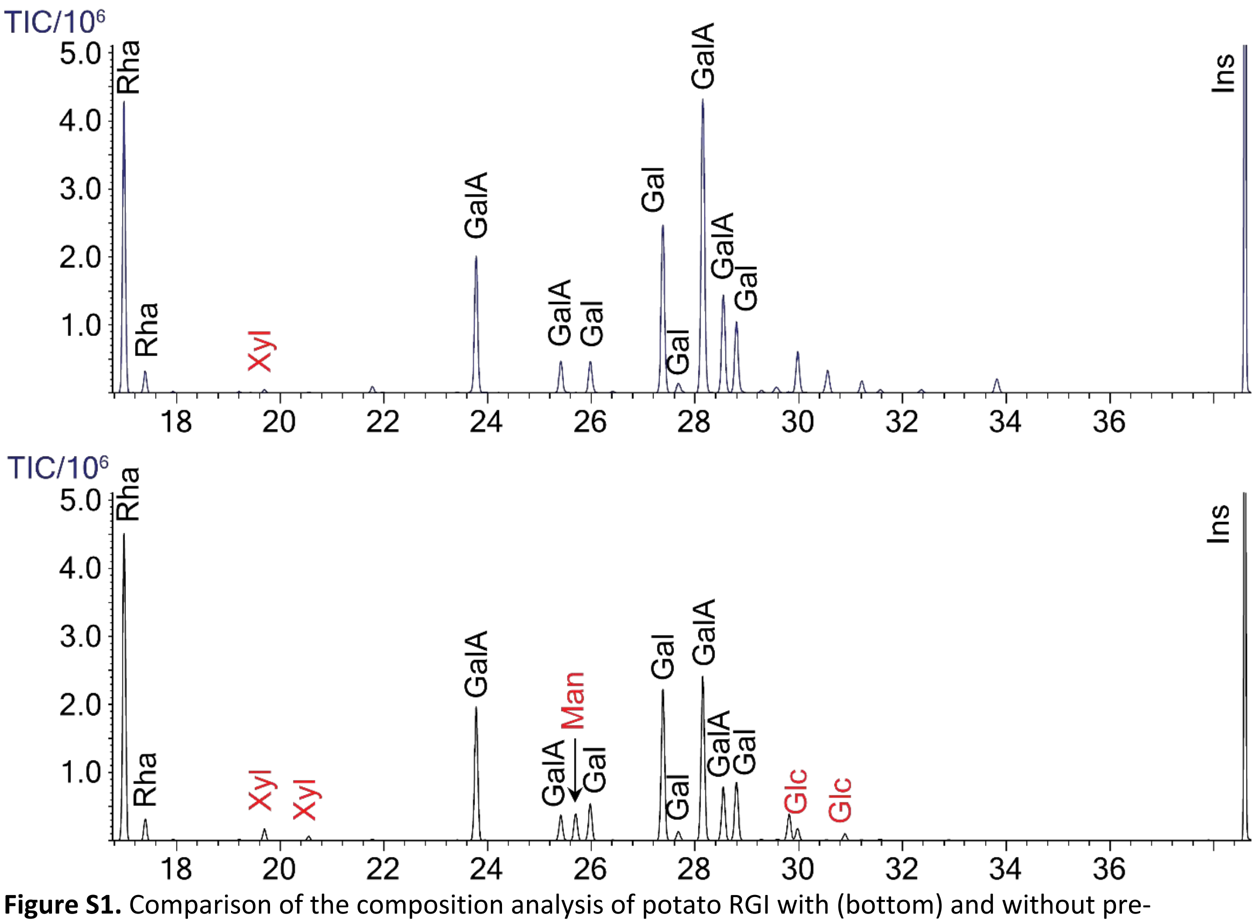

Figure S1. Comparison of the composition analysis of potato RGI with (bottom) and without pre-acetylation (top).

NMR Assignments

Table S1. Chemical shifts (ppm) and linkages of residues found in the sample of permethylated and reduced unsubstituted rhamnogalacturonan. For each group in a residue, the first row shows ^{1}H and the second one ^{13}C chemical shift.

Residue	Group											
	1	2	3	4	5	6	1-OMe	2-OMe	3-OMe	4-OMe	6-OMe	ROESY
**G**	5.00	3.60	3.60	4.23	4.31	–	–	3.43	3.46	–	3.35	R2
→4)-α-Gal-6d$_2$-(1→	95.7	76.9	79.8	70.4	68.4	70.2	–	57.3	57.7	–	58.9	
**R**	5.18	4.13	3.45	3.12	3.62	1.28	–	–	3.46	3.52		G4
→2)-α-Rha-(1→	98.1	73.0	80.4	81.6	68.5	17.9	–	–	57.7	60.6		
**A**	5.26	3.72	*3.47*	*3.13*	*3.62*	*1.28*	–	n.d.	n.d.	n.d.		
α-Rha-(1→	97.5	77.4	*79.9*	*81.8*	*68.5*	*17.9*	–	n.d.	n.d.	n.d.		
**B**	5.06	3.50	3.88	5.01	–	–	–	n.d.	n.d.		n.d.	E2
Δ^{4,5}-α-Gal-6d$_2$-(1→	97.3	77.6	74.1	97.5	–	–	–	n.d.	n.d.		n.d.	
**C**	5.04	3.62	3.65	4.22	*4.27*	–	–	n.d.	n.d.	–	n.d.	D2
4-α-Gal-6d$_2$-(1→	95.4	76.7	79.7	70.7	*68.6*	n.d.	–	n.d.	n.d.	–	n.d.	
**D**	4.65	4.01	3.46	3.13	3.54	1.30	3.35	–	n.d.	n.d.		
→2-α-Rha	98.0	72.0	80.0	81.4	68.1	17.9	54.8	–	n.d.	n.d.		
**E**	5.17	4.27	n.d.	n.d.	n.d.	n.d.	–	–	n.d.	n.d.		
→2-α-Rha-(1→	98.6	74.3	n.d.	n.d.	n.d.	n.d.	–	–	n.d.	n.d.		

Linkages (ROESY)
G1–R2
R1–G4
C1–D2
B1–E2

* Chemical shift assignments in italics are tentative.
n.d., not determined

Notes to NMR Table

Residue G is Gal-6d$_2$, resulting from the carboxyl reduction of the main RGI backbone GalA residues with $LiAlD_4$.

Residue R is the main RGI backbone Rha residue.

Residue D is likely Rha at the reducing end. The 1-O methyl group with distinct ^{13}C chemical shift was identified using ROESY and HSQC. The linkage between C1 and D2 was confirmed by ROE contacts between C1 and D2 as well as C1 and D1.

Residue C is identified as Gal-6d$_2$ in the penultimate position at the reducing end as shown by the ROE contacts between C1 and D2 and D1.

Residue B is the Δ^{4,5}-unsaturated α-Gal-6d$_2$ residue resulting from β-elimination of the next Rha residue from the GalA the methyl ester. It amounts to about 8% of the total GalA.

Residue E is the Rha next to the Δ^{4,5}-unsaturated α-Gal-6d$_2$ residue at the non-reducing end of those chains that have undergone β-elimination.

Residue A is Rha, based on interrupted spin–spin interactions in TOCSY. The downfield chemical shift displacement of A2 relative to R2 indicates that Residue A is methylated at the 2-position and not glycosylated and thus located at the non-reducing end. A1 shows an ROE with a position close to G4, indicating a linkage with a Gal-6d_2 residue. This residue might be the result of ring-opening during reduction[3] followed by loss of the $\Delta^{4,5}$-unsaturated α-Gal-6d_2 residue resulting from β-elimination.

In summary, we propose the presence of two types of polysaccharides, consisting of the residues listed in Table S1.

$$B–E–[G–R]_n–C–D$$

$$A–[G–R]_m–C–D$$

References

1 – Harris P. J.; Henry R.J.; Blakeney A.B.; Stone, B.A. *Carbohydr. Res.* **1984,** 127, 59-73.

2 – Anumula K. R.; Taylor P.B. *Anal. Biochem.* **1992**, 203(1), 101-108.

3 – Effenberger F.; *Angew. Chem. Int. Ed. Eng.* **1969** 8(5), 295-312.
